# Decreased levels of sex hormones in females with solitary pulmonary nodules were risk factors for malignancy

**DOI:** 10.1186/s13019-024-02609-x

**Published:** 2024-03-12

**Authors:** Wang Wendan, Li Mengyu, Zhang Qiufeng

**Affiliations:** Department of Internal Medicine, the Second Affiliated Hospital of Zhejiang University School of Medicine, HangZhou, Zhejiang China

**Keywords:** Solitary pulmonary nodules (SPNs), Sex hormones, Female, Malignancy risk factors, Logistic regression

## Abstract

**Objective:**

The purpose of this research was to detect the relationship between the levels of sex hormones in females with solitary pulmonary nodules (SPNs) and their potential malignancies.

**Methods:**

A total of 187 consecutive patients with pathologically confirmed SPNs by chest CT were enrolled in our study. They were divided into two groups based on the pathologic findings of SPNs after surgery: benign and malignant SPNs. Progesterone (P), estradiol (E2), and testosterone (T) levels in the two groups were measured. Meanwhile, we used binary logistic regression analysis to analyze the risk factors for SPNs.

**Results:**

Of these 187 patients, 73 had benign SPNs, while 114 had malignant SPNs. We found that the levels of progesterone (P), estradiol (E2), and testosterone (T) were decreased significantly in patients with malignant SPNs compared to patients with benign SPNs (all *P* < 0.05). Multivariate logistic regression analysis revealed that second-hand smoke, burr sign, lobulation sign, pleural traction sign, vascular convergence sign, vacuole sign, and ≥ 1 cm nodules were independent risk factors for malignant pulmonary nodules in females.

**Conclusions:**

Decreased levels of sex hormones in females were associated with malignant pulmonary nodules, suggesting that they can contribute to the diagnosis of lung cancer.

Lung cancer is a malignant tumor originating from bronchial epithelial cells. Studies have shown that lung cancer is the second leading cause of cancer-related deaths, even if there is a decrease in incidence and mortality each year [1, 2]. It is classified into two main histologic subtypes, small cell lung cancer and non-small cell lung cancer, with the latter accounting for almost 80% [3, 4]. Data show a higher incidence of lung cancer in females than in males [5]. As an early important manifestation of lung cancer, solitary pulmonary nodules play an important role in early warning. Fortunately,, only a small number of pulmonary nodules eventually confirmed lung cancer [6]. Therefore, accurate judgment of pulmonary nodules and timely surgical intervention are of great significance for improving the 5-year survival rate of patients. For patients with non-high-risk pulmonary nodules, dynamic follow-up can save medical resources and reduce the burden of those patients.

Recently, a variety of lung cancer-related markers have been presented, but few have eventually turned out to have clinically high sensitivity and specificity for the early diagnosis of lung cancer[7]. It has been affirmed that sex hormones influence almost all aspects of normal human physiological functions, cell activities, and behaviors, especially in females[8]. Abnormal expression of sex hormones is thought to play an important role in the etiology of some cancers, such as breast cancer, cervical cancer and ovarian cancer.

At the same time, some scholars have observed the abnormal expression of sex hormones in the peripheral serum of patients with lung cancer, which was initially confirmed in male patients, but less research has been conducted in female patients [9]. In addition, due to the lack of specific performance for early pulmonary nodules, CT scans are currently the main means of detecting lung cancer. Therefore, some signs of CT are also the main hints of malignant transformation of pulmonary nodules[10]. Based on the above theory, we used a logistic regression model to predict the benign and malignant probability of pulmonary nodules in females.

## Methods patients and clinical samples

A total of 187 consecutive female patients of SPNs, diagnosed by chest CT and confirmed by pathology, were collected in our study. Inclusion criteria: (1) meet the diagnostic criteria for solitary pulmonary nodules: a single spherical lesion that is capable of being accurately measured to permit accurate measurement of its size (3 cm or less in diameter); (2) Patients with benign or malignant tumors confirmed by clinical pathology or biopsy; exclusion criteria: (1) Preoperative imaging confirmed the presence of bone metastasis or brain metastasis; (2) Patients with previous gynecological tumors and breast lesions; (3) Patients with a history of pituitary adenoma; (4) Heart, kidney and other major organ dysfunction.

## Radiological examinations

Low-dose spiral CT: Using Philips Brilliance64-row spiral CT scanner, pulmonary (apical to renal hilar) fluid was performed Flat) Low dose scanning, scanning parameters set: current 120 mA, voltage 120 kV, lung window image window-700 HU, window width 1500 HU, mediastinal image window 50 HU, window width 350 HU, scanning thickness 5 mm, the interval between layers was 5 mm, and the lung window was reconstructed at 1.5 mm.

The chest low-dose CT images of all patients were examined by two radiologists (a deputy chief physician and an above-level physician) with at least ten years of relevant experience. The size of lesions, glitches, air cavity density (including vacuole sign and inflatable bronchioles), calcification, pleural indentation, satellite lesions, and vascular aggregation were examined. For those patients with inconsistent results, the final results were achieved after discussion.

## Measurement of serum levels of testosterone, estradiol, and progesterone

The fasting peripheral venous blood of the two groups of subjects was routinely extracted, and approximately 3–5 ml was added to the anticoagulant tube. Then, it was put into the centrifuge and centrifuged at a speed of 3000 r/min, and the supernatant was separated and stored at − 80 °C for later analysis. The contents of testosterone, estradiol, and progesterone were detected and monitored by an enzyme-linked immunosorbent assay kit (the kit was provided by Mingde Biotechnology Co., Ltd., Wuhan, Hubei, and the item numbers: YB-I0012,10902). The specific operation method was performed according to the standard method of the ELISA kit.

### Statistical analysis

SPSS Statistics version 24.0 (SPSS, Chicago, IL, USA) was utilized for data analysis. The characteristics of the patient are given as the median with interquartile range (IQR). Differences between groups and relationships between different indices were analyzed using nonparametric tests. The incidence of variables was compared using Fisher’s exact test or χ2 test in contingency tables. Variables with statistically significant differences and prior research recommendations were chosen through univariate analysis screening. The indicators related to the risk of malignant pulmonary nodules were included in the logistic multivariate regression analysis, the risk factors and their coefficients were obtained, and a malignant pulmonary nodule prediction model was established. Multiple regression logistic regression was performed to evaluate the risk relationship between levels of sex hormones and malignant pulmonary nodules. For all analyses, *P*<0.05 was considered statistically significant.

## Result

### Patient baseline characteristics

A total of 187 female patients who met our selection criteria were recruited from the respiratory medicine clinic of our hospital. Patients with benign or malignant tumors were confirmed by clinical pathology or biopsy. Of 187 cases, 74 had benign SPNs, while 113 had malignant SPNs. No statistically significant differences were observed in the baseline characteristics of benign and malignant SPNs (all *P* > 0.05). The exposure rate of secondhand smoke in the benign group was higher than that in the malignant group (*P* < 0.05). Table 1.

**Table 1:** Characteristics of the Cohort

Group	numbers	Age(years)	BMI(kg/m2)	Menstrual cycle	Parity	Menopause	Second-smoker
Benign SPNs	73	57.8(17)	23.8(4.0)	27.4(3.1)	2.2(1.0)	15()	18
Malignant SPNs	114	58.0(16)	24.0(4.2)	27.9(3.2)	2.3(1.2)	27()	49
Z/χ2 value	-	0.670	0.563	0.439	0.823	0.251	6.500
*P* value	-	0.433	0.774	0.561	0.275	0.616	0.011

Continuous variables are presented as the mean ± standard deviation or median (interquartile range). Categorical variables are given as n (%)

### Clinical characteristics of malignant nodules

In 114 cases of malignant pulmonary nodules, there were 89 cases of lung adenocarcinoma, 12 cases of lung squamous cell carcinoma, 8 cases of large cell lung cancer, 3 cases of small cell lung cancer and 2 cases of others. Table 2.

**Table 2:** Clinical characteristics of 114 malignant SPNs

Variable	Numbers	Constituent ratio(%)
NSCLC adenocarcinoma	78	68.42
squamous cell carcinoma	12	10.53
large cell lung cancer	8	7.02
others	2	1.75
SCLC	14	12.28

NSCLC: non-small cell lung cancer; SCLC: small cell lung cancer

### Comparison of imaging data of patients with benign and malignant pulmonary nodules

Univariate analysis revealed statistically significant differences in the burr sign, lobulation sign, pleural traction sign, vascular convergence sign, vacuole sign, size, calcification, and satellite lesions between benign and malignant SPNs. However, there were no distinct changes between groups for the location and type of nodule. Table 3.

**Table 3:** Univariate Analysis of malignant SPNs (n, %)

CT signs	Benign SPNs (*n* = 73)	Malignant SPNs (*n* = 114)	F/χ2 value	P value
Location left lung	54	76	0.0176	0.894
right lung	19	28		
Burr sign	16	42	4.633	0.031
Lobulation sign	18	45	4.373	0.037
Pleural traction sign	19	51	6.651	0.010
Vascular convergence sign	12	40	7.710	0.005
Vacuole sign	13	48	11.935	0.000
Size >1 cm	16	55	13.097	0.000
≤1 cm	57	59		
Type of nodule solid nodules	28	58	2.131	0.144
ground glass nodule	35	44		
mixed nodules	10	12		
Calcification	28	9	26.018	0.000
Satellite lesions	19	10	10.113	0.001

### Serum levels of sex hormones in female patients with benign and malignant SPNs

**Fig. 2:**
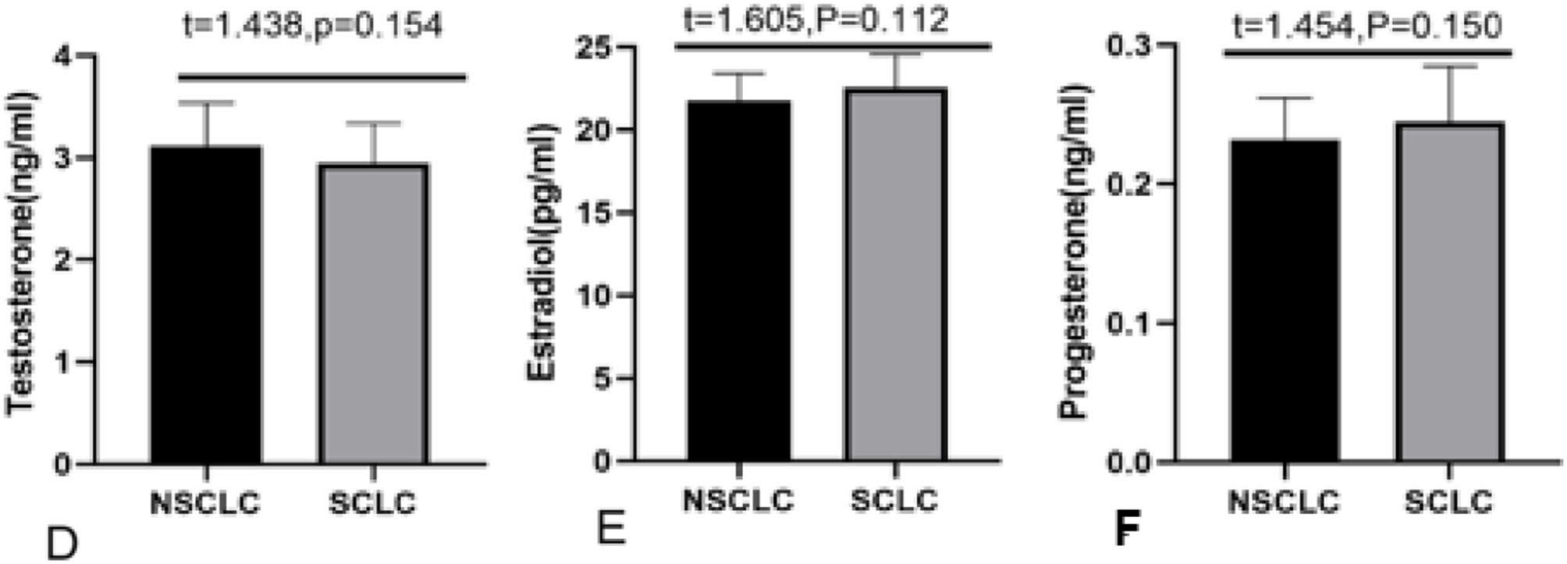
NSCLC: non-small cell lung cancer. SCLC: small cell lung cancer

In our study, we measured sex hormones (testosterone, estradiol, and progesterone) in the serum of all female patients. Statistical analysis showed that the levels of testosterone, estradiol, and progesterone(3.227 ± 0.59 ng/mlvs4.812 ± 0.58 ng/ml,22.66 ± 2.45 pg/mlvs32.63 ± 2.65 pg/ml,0.250 ± 0.06 ng/ml vs0.460 ± 0.04 ng/ml, respectively) in the peripheral serum of patients in the benign group were statistically significantly higher than those in the malignant group(all*P*<0.05). We further analyzed the expression of sex hormones in patients with different types of lung cancer. The results showed that there was no significant difference in testosterone, estradiol, or progesterone (3.116 ± 0.42 ng/ml vs. 2.945 ± 0.39 ng/ml, 21.77 ± 1.63 pg/ml vs. 22.54 ± 2.03 pg/ml, 0.232 ± 0.03 ng/ml vs. 0.245 ± 0.04 ng/ml, respectively) between small cell lung cancer and non-small cell lung cancer (all *P* > 0.05). Figure 1.&Figure 2.

**Figure:**
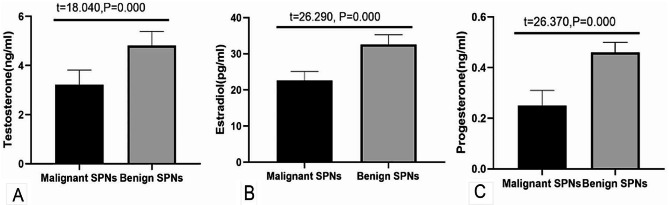
Figure 1

### Multivariable analysis determining covariate factors associated with malignant SPNs

Multivariate logistic regression analysis was used to detect the potential protective and risk factors in the progression of malignant SPNs. Table 4 shows that calcification and satellite lesions were protective factors against malignant SPNs. The burr sign, lobulation sign, pleural traction sign, vascular convergence sign, vacuole sign and size were independently correlated with malignant SPNs (Table 4).

**Table 4:** Multivariable analysis determining covariate factors associated with malignant SPNs

	All eligible subjects(*n* = 187)			
Variable	standardizedβ	OR	95%CI	P value
Burr sign	0.773	3.57	1.58–8.90	0.040
Lobulation sign	0.658	3.43	1.25–7.35	0.032
Pleural traction sign	0.547	4.01	1.04–7.59	0.021
Vascular convergence sign	0.376	4.52	1.37–9.04	0.001
Vacuole sign	0.554	3.64	1.05–10.02	0.023
Size>1.0 cm	0.738	3.82	1.14–8.77	0.004
Calcification	-3.211	0.54	0.34–0.65	0.045
Satellite lesions	-4.217	0.76	0.43–0.75	0.003
Second-hander	0.534	4.59	1.57–10.49	0.001
Serum levels of testosterone	0.432	2.31	1.09–5.13	0.037
Serum levels of estradiol	0.451	2.02	1.28–4.63	0.001
Serum levels of progesterone	0.399	2.48	1.33–4.25	0.029

## Discussion

According to the latest data from the American Cancer Society in 2022, lung cancer is the most common malignant tumor, with the highest mortality rate in men (22%) and the second highest mortality rate in women (17%), second only to breast cancer (18%)[11, 12]. Due to the insidious onset of lung cancer in patients, the clinical symptoms are not obvious, so the best surgical treatment is missed. In addition, the early manifestations of lung cancer are mostly solitary nodules[13]. Due to the different shapes and sizes of nodules, the probability of deterioration cannot be accurately judged, which poses a great challenge to clinicians. Previous literature has confirmed that the incidence of lung cancer in women is higher than that in men[14]. Based on one of the conclusions combined with the differential expression of sex hormones between sexes, we assume that sex hormones should be differentially expressed in tumors of different natures, which is also the purpose of this study.

In this study, the results of 187 cases of solitary pulmonary nodules showed that the malignancy rate was 60.96%, which was similar to the previous results of female pulmonary nodules[15, 16]. We further analyzed the pathological types and revealed that NSCLC accounted for 87.72%, and lung adenocarcinoma was the main pathological type, once again confirming that NSCLC is the main pathological type of lung cancer and that there is a high incidence of adenocarcinoma[17, 18].

### Chest low-dose CT scan of solitary pulmonary nodules

Since the 1990s, with the development of chest low-dose CT technology, lung cancer screening has entered the era of low-dose CT. Foreign lung cancer screening tests such as the International Early Lung Cancer Action Plan (I-ELCAP), the National Lung Cancer Screening Test (NLST), and the Netherlands-Belgium Lung Cancer Screening Study (NELSON) have confirmed that low-dose chest CT scans can significantly improve the early detection rate and survival rate of lung cancer and reduce the mortality of lung cancer[19–21].

Early CT scans of pulmonary nodules are an important screening method and current diagnosis method for early lung cancer. We analyzed general data and CT signs in our study. There were no statistically significant differences in the location and type of nodule, but statistically significant differences were observed for the burr sign, lobulation sign, pleural traction sign, vascular convergence sign, vacuole sign, size, calcification, and satellite lesions[22, 23]. The pathological basis of burr signs is the infiltration of tumor cells into adjacent bronchial vascular sheaths or local lymphatic vessels or the fibrous bands of tumor-promoting connective tissue formation. On CT imaging, a radial nonbranched short linear shadow extending from the edge of the lung mass or nodule to the parenchyma around the lung and not connected to the pleura was present. The lobulation sign is mainly due to the uniform density of peripheral lung cancer. The pleural traction sign is mainly attributed to nodules pulling the pleura. The vascular convergence sign mainly manifests as the composition of thickened blood vessels, mainly because the rapid growth of nodules requires a greater blood supply. The vacuole sign is due to residual air-containing lung tissue or bronchial formation in the lung mass, a 1-3 mm air-containing low-density area. There is a direct correlation between the size of pulmonary nodules and the probability of malignancy. It has been reported that the malignant degree of nodules with a maximum diameter of 1 cm is higher than that of benign nodules, mainly because the growth rate of malignant nodules is greater than that of benign nodules. Notably, our results showed that the Burr sign, lobulation sign, pleural traction sign, vascular convergence sign, vacuole sign, and size were independent risk factors for malignant SPNs. Calcification and satellite lesions are protective factors for the deterioration of pulmonary nodules because most of them are benign lesions, and most malignant lesions have no such manifestations. Finally, by analyzing the baseline data, the author shows that secondhand smoke is an important exposure factor for lung cancer and suggests that women should try to avoid tobacco. The above conclusions are consistent with the conclusions of previous scholars[24, 25].

### Serum levels of sex hormones in malignant SPNs

Previous studies have shown that under normal circumstances, both androgens (testosterone) and estrogens (estradiol, progesterone) are considered to have beneficial effects on regulating lung structure and function [26, 27]. Once a tumor develops in the lungs, the sex hormone levels in the body will be disrupted. The results of this study show that testosterone, estradiol. The progesterone content in peripheral blood of benign group was significantly higher than that of malignant group. The potential mechanisms include: sex hormone promotes tumor cell proliferation through its downstream receptor (ER/MAPK) and prevents lung cancer cell apoptosis[28]. In addition, estrogen may promote the proliferation of hormone-dependent tumor cells through the following mechanisms: (1) regulate the expression of early genes related to cell division, such as c-myc, c-jun and c-fos, and promote DNA synthesis and division; Stimulate the expression of growth factors, such as epidermal growth factor (EGF), insulin-like growth factor I II (IGF-II) and transforming growth factor-A (TGF-a) to promote cell proliferation; (3) Regulate the expression of regulatory proteins related to cell cycle, such as cyclin-B2, and affect cell proliferation. In addition, sex hormones can also participate in the formation of lung cancer in other ways, mainly including: (1) Regulate the expression of regulatory proteins related to cell cycle; (2) Inhibiting the lipid peroxidation ability of oxygen free radicals leads to the reduction of malondialdehyde (MDA), the metabolite of oxygen free radicals, which can inhibit the growth and metabolism of cells, resulting in abnormal and vigorous cell metabolism and enhanced mitosis; (3) Sex hormones spread into the cytoplasm through the cell membrane and reach the nucleus, bind to the specific site of chromatin in the nucleus, cause nuclear protein allosterism, thus activating and affecting DNA transcription, inducing abnormal proteins or enzymes, causing biological effects that lead to changes in the target cell quality and quantity, and promoting the growth of cancer cells. At the same time, the use of hormone therapy for patients with lung cancer has gradually become a treatment method in clinical practice, which has again confirmed that there is a certain correlation between sex hormones and the occurrence of lung cancer. However, whether there is a correlation between sex hormone levels and pathological types of lung cancer still needs further study.

### Study limitations

First, this study is a retrospective study. The subjects were patients who were more likely to have malignant nodules and were willing to undergo surgical resection, and there was selection bias. The conclusion needs to be further confirmed by a large sample size, multicenter, prospective clinical study. In addition, the threshold for the diagnosis of pulmonary nodules by serum sex hormones also needs to be accurately determined.

## Conclusions

Decreased levels of sex hormones in females were associated with malignant pulmonary nodules, as well as the Burr sign, lobulation sign, pleural traction sign, vascular convergence sign, vacuole sign, and size. Clinicians should combine CT scan results and serum blood test results to make accurate diagnoses of pulmonary nodules.

## Data Availability

Not applicable.
